# Regulation and coordination of the different DNA damage responses in *Drosophila*

**DOI:** 10.3389/fcell.2022.993257

**Published:** 2022-09-06

**Authors:** Antonio Baonza, Sara Tur-Gracia, Marina Pérez-Aguilera, Carlos Estella

**Affiliations:** Centro de Biología Molecular “Severo Ochoa”, CSIC-UAM, Madrid, Spain

**Keywords:** DNA damage response, apoptosis, *Drosophila*, cell cycle, *p53*, tissue homeostasis, cellular context, ionizing radiation

## Abstract

Cells have evolved mechanisms that allow them to respond to DNA damage to preserve genomic integrity and maintain tissue homeostasis. These responses include the activation of the cell cycle checkpoints and the repair mechanisms or the induction of apoptosis that eventually will eliminate damaged cells. These “life” vs. “death” decisions differ depending on the cell type, stages of development, and the proliferation status of the cell. The apoptotic response after DNA damage is of special interest as defects in its induction could contribute to tumorigenesis or the resistance of cancer cells to therapeutic agents such as radiotherapy. Multiples studies have elucidated the molecular mechanisms that mediate the activation of the DNA damage response pathway (DDR) and specifically the role of p53. However, much less is known about how the different cellular responses such as cell proliferation control and apoptosis are coordinated to maintain tissue homeostasis. Another interesting question is how the differential apoptotic response to DNA damage is regulated in distinct cell types. The use of *Drosophila melanogaster* as a model organism has been fundamental to understand the molecular and cellular mechanisms triggered by genotoxic stress. Here, we review the current knowledge regarding the cellular responses to ionizing radiation as the cause of DNA damage with special attention to apoptosis in *Drosophila*: how these responses are regulated and coordinated in different cellular contexts and in different tissues. The existence of intrinsic mechanisms that might attenuate the apoptotic pathway in response to this sort of DNA damage may well be informative for the differences in the clinical responsiveness of tumor cells after radiation therapy.

## Introduction

Eukaryotic cells employ a diverse array of responses that include cell cycle arrest and DNA repair mechanisms to preserve genomic integrity after DNA damage. However, if the damage is too severe or it cannot be repaired, cells can activate the programed cell death pathway or apoptosis to prevent the transmission of defective DNA material to the daughter cells (Jackson and Bartek, 2009).

In response to DNA damaging agents such as ionizing radiation (IR), cells trigger the DNA damage response (DDR) pathway that consists in an evolutionary conserved set of proteins that function as sensors, transducers and effectors of the genotoxic stress (Marechal and Zou, 2013). Mutations in many of the genes that encode for members of this pathway have been linked to a variety of diseases including cancer-predisposition (Ciccia and Elledge, 2010). DNA damage activates a coordinated response that includes cell cycle checkpoint and apoptotic induction (Song, 2005; Blackford and Jackson, 2017). The tumor suppressor p53 plays a central role in the coordination of all these cell decisions (Vousden and Lane, 2007). p53 activity is tightly regulated by post-translational modifications, cofactor interactions, cell proliferating status and developmental and cellular context that can influence p53 cell fate decision making (Bode and Dong, 2004; Lunardi et al., 2010; Hafner et al., 2019). Other effectors of the DDR pathway are the E2f transcription factors and the c-Jun N-terminal kinase (JNK) that mediate some of the cellular responses, such as apoptosis (Stevens et al., 2003; Picco and Pages, 2013).

The use of *Drosophila* as a model organism to study the DNA damage pathway has been proven to be very valuable to decipher the cellular and molecular mechanisms that control and coordinate the different DNA damage induced responses such as cell cycle arrest, DNA repair, senescence, apoptosis and radioresistance (Song, 2005; Khan et al., 2019). Notably, 65% of human genes related to diseases have homologs in the fly (Chien et al., 2002). The short generation time, the powerful genetic tools available and the low genetic redundancy makes *Drosophila* an ideal organism to model human disease mechanisms.

Here we review the different mechanisms that are involved in the cellular response induced by IR. We especially focus on the mechanisms that trigger the apoptotic response. We also review how this response is regulated in different developmental and cellular contexts in *Drosophila*.

## Apoptotic response to irradiation-induced DNA damage in *Drosophila*

**FIGURE 1:**
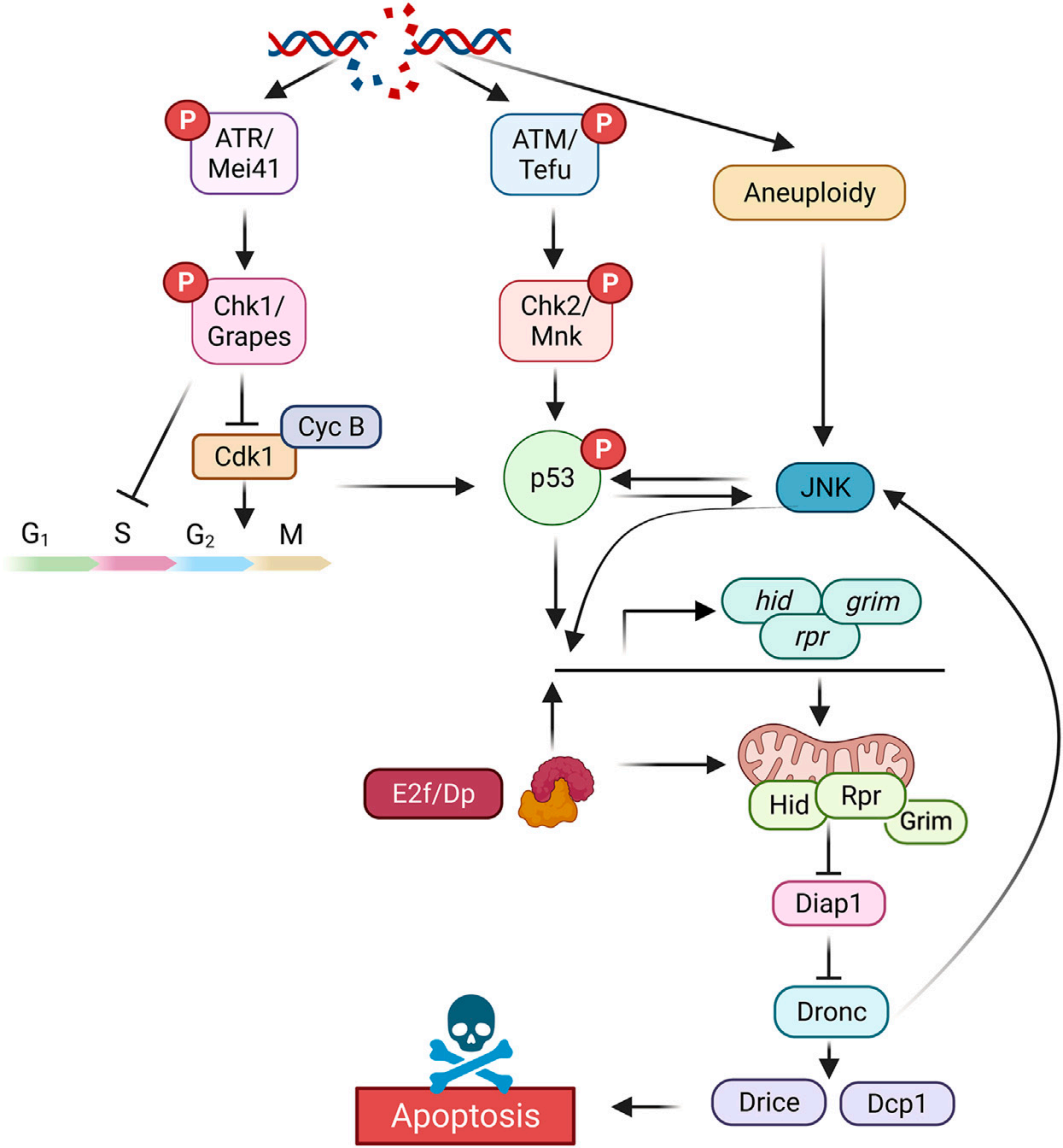
Simplified representation of the different components of the DNA damage response pathway that leads to cell cycle arrest and apoptotic induction in *Drosophila*. Created with BioRender.com.

In *Drosophila* the presence of double strand breaks (DSB) is detected by sensors of DNA lesions such as the MRE11–RAD50–NBS1 (MRN) protein complex. These proteins activate the upstream kinases ATM (ataxia-telangiectasia mutated or Telomere fusion in *Drosophila* (Tefu)), and ATR (ATM- and Rad3-Related or Meiotic-41 in *Drosophila* (Mei41)) (Marechal and Zou, 2013). Activated ATM/Tefu and ATR/Mei41 phosphorylate a number of substrates, such as the downstream kinases Chk1/Grapes (Grp) and Chk2/Mnk, which regulates cell cycle arrest, DNA repair and apoptosis (Brodsky et al., 2004; Jaklevic and Su, 2004; Song et al., 2004; de Vries et al., 2005; Song, 2005; Khan et al., 2019). While the ATR/Chk1 branch of the pathway mostly controls cell cycle arrest and DNA repair, the function of the ATM/Chk2 axis is required for *p53*-mediated apoptotic induction (Xu et al., 2001; Peters et al., 2002; Brodsky et al., 2004; Jaklevic and Su, 2004; Song et al., 2004; Bi et al., 2005; Khan et al., 2019) (Figure 1). A single *p53* ortholog, compare to the three mammalian members (*p53*, *p63*, and *p73*) has been identified in *Drosophila*, which makes its study easier (Brodsky et al., 2000a; Ollmann et al., 2000). Contrary to its mammalian ortholog, *Drosophila p53* regulates apoptosis but not cell cycle arrest after irradiation (Brodsky et al., 2000a; Jin et al., 2000; Ollmann et al., 2000; Lee et al., 2003; Sogame et al., 2003). This cell cycle checkpoint is mediated by the ATR/Mei41 and Chk1/Grapes axis that transiently downregulates Cdk1 active levels and therefore arrests cells at the G2/M transition (Hari et al., 1995; Brodsky et al., 2000b; de Vries et al., 2005; Jin et al., 2008; Ayeni et al., 2014). In addition, other checkpoints, such as a S phase entry delay (intra-S checkpoint) have been described in other tissues like the larval brain (Jaklevic and Su, 2004; Song, 2005) (Figure 1).

DNA damage induces Chk2 dependent activation of p53, which is necessary for timely apoptotic induction through the regulation of the proapoptotic genes *rpr*, *hid*, and *grim* (Brodsky et al., 2000a; Peters et al., 2002; Lee et al., 2003; Sogame et al., 2003; Brodsky et al., 2004; Akdemir et al., 2007) (Figure 1). p53 responding elements (p53^REs^) have been identified for both *rpr* and *hid* genes where p53 directly regulates their transcription (Brodsky et al., 2000a; Zhang et al., 2014). Interestingly, in response to DNA damage the *rpr* p53^RE^ could make long-range chromatin contacts in *cis* and *trans* to regulate the expression of other cell death genes at distant positions, including *hid*, and *sickle* (*skl*) (Link et al., 2013). Deletion of the *rpr* p53^RE^ not only abolish DNA damage induction of *rpr*, but also of other apoptotic genes such as *hid* and *skl*. However, although the binding of p53 to the *rpr* p53^RE^ is not a prerequisite for these chromatin contacts to occur, it is required for the transcriptional activation of the apoptotic genes after irradiation (Link et al., 2013). From all the apoptotic genes, at least in imaginal discs, *hid* seems to play the major role for IR-induced cell death, since *hid* mutant discs show near complete depletion of apoptosis 4 h after irradiation (McNamee and Brodsky, 2009).

When the levels of expression of the apoptotic factors have reached a threshold, they promote the inhibition of *Drosophila* apoptosis protein 1 (Diap1). Diap1 functions as an inhibitor of the initiator caspase Dronc, an ortholog of *caspase 9*, and of the effector caspases Drice and Dcp1. Therefore, Diap1 degradation leads to Dronc activation, which promotes cell death [reviewed in Steller (2008)] (Figure 1).

As it is show below, at longer times after irradiation, a p53-independent cell death program is also activated for the elimination of cells with damaged DNA (Wichmann et al., 2006). In *p53* and *Chk2* mutant wing discs, IR-induced expression of the proapoptotic genes and cell death is still observed, although at lower levels and later time points when compared to the wild type controls (Wichmann et al., 2006). Therefore, while p53 is required for the rapid elimination of cells with damaged DNA during the first 18 h after irradiation, a p53-independent mechanism maintains genomic integrity at later times.

In addition to the activation of the apoptotic genes, p53 has been shown to be required for the regulation of the Hippo and JNK pathway that also contributes to cell death regulation after DNA damage. After irradiation, the Hippo pathway is activated in a p53 dependent manner, decreasing the levels of Diap1, and therefore promoting apoptosis (Colombani et al., 2006). Both in mammals and in *Drosophila*, the JNK signaling plays an important role in IR-induced apoptosis (McEwen and Peifer, 2005; Picco and Pages, 2013). In irradiated wing discs, p53 is necessary to induce JNK activation as revealed by the expression of *puckered* (*puc*), a JNK target gene that encodes for a phosphatase that acts in a negative feedback loop to limit JNK signaling. Downregulation of the JNK pathway by overexpressing *puc*, significantly reduces IR-induced cell death (McEwen and Peifer, 2005). This apoptosis regulation by the JNK pathway seems to be mediated by the proapoptotic genes *rpr* and *hid* (McEwen and Peifer, 2005; Luo et al., 2007) (Figure 1). Accordingly, in the eye retina, the transcriptional effectors of this pathway, Kay/Fos, directly regulate the expression of the gene *hid* in response to ultraviolet (UV) irradiation (Luo et al., 2007). The apoptotic function of JNK seems to be ligand independent, as mutants for *eiger*, the unique Tumor necrosis factor (TNF) ligand identified in *Drosophila*, do not show a reduction in IR-induced apoptosis in the wing disc (Brodsky et al., 2004). All these together suggest that in response to DNA damage, JNK could act downstream of p53 and contribute to the induction of cell death. Interestingly, this apoptotic response is amplified by a positive loop mediated by the apical caspase Dronc that, downstream of the initial activation of the proapoptotic genes, triggers *p53* and JNK pathway (Wells et al., 2006; Shlevkov and Morata, 2012). Moreover, the JNK pathway and p53 can also activate each other independently of caspase activation providing an effective and robust apoptotic response to DNA damage (Gowda et al., 2012; Shlevkov and Morata, 2012; Sanchez et al., 2019) (Figure 1).

In addition to *p53* and JNK, the E2f transcription factors also contribute to the early elimination of cells with damaged DNA through apoptotic regulation. The E2f proteins play an essential role in the control of cell cycle progression and apoptosis. Compared to the mammalian E2f family, *Drosophila* encodes for only two E2Fs, an activator E2f1 and a repressor E2f2, a single obligatory cofactor named Dp and two E2f repressors, the retinoblastoma (Rb) family members Rbf1 and Rbf2 (van den Heuvel and Dyson, 2008). The use of viable *E2f1* alleles and *Dp* mutants has revealed the contribution of these factors to apoptotic induction after DNA damage. While E2f1/Dp promote apoptosis in response to DNA damage in regions of active cell proliferation, such as the intervein regions of the wing disc, these genes protect cells from IR-induced apoptosis in domains containing cycle arrested cells, such as the zone of nonproliferation cells (ZNC) in the dorsal/ventral (D/V) boundary (Moon et al., 2005). In the D/V boundary of non-irradiated discs *hid* expression is upregulated in *Dp* mutants by an unknown factor, suggesting that E2f1/Dp can attenuate IR-induced apoptosis in these cells by repressing *hid*. A direct binding of E2f1 and to a lesser degree of E2f2 was found at the promoter of *hid* that could mediate this apoptotic regulation (Moon et al., 2005; Tanaka-Matakatsu et al., 2009). These results highlight the role of E2f1/Dp factors in regulating the apoptotic response to DNA damage in a cellular context dependent manner (Figure 1).

Both p53 and E2f1 seem to cooperate in the induction of cell death in the early response after irradiation (4 h post-IR), as the absence of p53 or E2f1/Dp strongly suppressed DNA-damage induced apoptosis, both in the wing and the eye imaginal discs (Moon et al., 2005; Moon et al., 2008). Although both proteins are able to bind and regulate the expression of *rpr* and *hid* (Brodsky et al., 2000a; Moon et al., 2005), only p53 is absolutely required for the induction of the apoptotic genes in response to DNA damage (Brodsky et al., 2000a; Jin et al., 2000; Peters et al., 2002; Sogame et al., 2003; Moon et al., 2008). While in vertebrates E2f1 proapoptotic activity is stimulated by DNA damage through E2f1 phosphorylation by the ATM/ATR kinases, it is not known if a similar mechanism operates in *Drosophila* (Lin et al., 2001; Pediconi et al., 2003).

Importantly, it has been shown that in *Dp* mutants, a condition that abolishes all E2f1 and E2f2 function, the apoptotic pathway must be blocked downstream of the expression of the proapoptotic genes *rpr* and *hid*, as both genes are strongly upregulated after irradiation, even though apoptosis is no detected (Moon et al., 2008; Ambrus et al., 2013).

Differential transcriptional analysis of wild type and *Dp* mutants discs exposed to IR and chromatin immunoprecipitation (ChIP-seq) experiments have shown a direct regulation of mitochondria-associated genes by the E2f/Dp transcriptional factors (Ambrus et al., 2013) (Figure 1). The mitochondria is a central regulator of apoptosis and it has been shown that the proapoptotic proteins Rpr, Hid, and Grim localized in this organelle (Haining et al., 1999; Claveria et al., 2002; Olson et al., 2003; Clavier et al., 2016). Functional studies demonstrated that mitochondrial function is severely compromised in *Dp* mutants and that downregulation of key E2f/Dp mitochondria target genes such as *Mdh2* or *ND42* have a strong protection on irradiated-induced apoptosis (Ambrus et al., 2013). How the mitochondrial defects observed in *Dp* mutants could affect the ability of the proapoptotic genes to trigger apoptosis is an interesting question to be addressed. In summary, the E2f/Dp pathway could regulate irradiated-induced apoptosis at multiple levels of the apoptotic cascade.

### DNA damage induced p53-independent apoptosis

Although p53 is considered the main effector of apoptotic induction in response to DNA damage, multiple reports have shown, both in *Drosophila* and in vertebrates, a delayed but still present apoptotic response in *p53* mutant cells (Strasser et al., 1994; Merritt et al., 1997; Wichmann et al., 2006; McNamee and Brodsky, 2009; Wichmann et al., 2010; Wells and Johnston, 2012). As we have mentioned before, in the absence of p53 or Chk2 a p53-independent apoptosis becomes detectable gradually at later time points (around 18 h post-IR) although lower than in wildtype cells at the same time points after irradiation (Wichmann et al., 2006). This p53-independent cell death is observed after cells have reentered into the cell cycle after the initial checkpoint and depends on the induction of the apoptotic genes, at least of *hid*, and on caspase activation (Wichmann et al., 2006; McNamee and Brodsky, 2009; Wichmann et al., 2010). Therefore, p53 seems to be responsible for the fast elimination of cells with damaged DNA through the canonical DDR pathway. Nevertheless, a later p53-independent apoptotic mechanism eliminates IR-induced defective cells. The generation of aneuploid cells by irradiation could trigger this p53-independet apoptosis, though the molecular mechanisms that link these cells to the activation of the apoptotic cascade are unknown (McNamee and Brodsky, 2009; Dekanty et al., 2012).

Again, the E2f/Dp family and the JNK pathway also contributes to this p53-independent apoptosis. Wing imaginal discs from *E2f1* and *Chk2* or *E2f1* and *p53* double mutants show reduced *hid cis*-regulatory module activation and decreased apoptosis 24 h after irradiation when compared to single *p53* or *Chk2* mutants (Wichmann et al., 2010). The apoptotic function of E2f1 seems to be relevant only in *p53* mutant cells, as robust apoptosis is observed in single *E2f1* mutant discs at this time points (24 h post-IR) (Wichmann et al., 2010). As E2f1 plays an important role regulating G1/S phase transition, and cell proliferation has been shown to influence both p53-dependent and -independent apoptosis, it is possible that the effect of E2f1 on IR-induced apoptosis could be an indirect effect due to altered cell proliferation (Ruiz-Losada et al., 2022). The other E2f *Drosophila* member, E2f2, behaves as a repressor of IR-induced apoptosis during the p53-independent phase. Consistently with its repressor function, *p53 E2f2* double mutants have significantly more IR-induced apoptosis than single *p53* mutants (Wichmann et al., 2010). These results indicate that the two *Drosophila* E2f family members, E2f1 and E2f2, have opposing functions on the regulation of p53-independent apoptosis: E2f1 contributes to cell death after irradiation, while E2f2 restrains it in the absence of p53 (Wichmann et al., 2010). The regulation of p53-independent apoptosis resembles the molecular logic of *PCNA* regulation, where E2f1 activates and E2f2 represses *PCNA* expression through canonical E2f sites (Frolov et al., 2001). In the complete absence of E2f function, such as in *Dp* mutants, p53-independent apoptosis is maintained, suggesting that other factors may contribute to the induction of cell death (Wichmann et al., 2010). This result is surprising due to the previously described role of E2f/Dp regulating mitochondria function and apoptotic induction in early-irradiated eye imaginal discs (Ambrus et al., 2013). It is possible that p53-independent apoptosis does not depend on the mitochondria and that the E2f transcription factors have a more direct role regulating proapoptotic gene expression. It has been pointed out that the pro-apoptotic and anti-apoptotic role of E2F/Dp proteins depends on the presence of p53. As p53 is a more potent apoptotic inducer than E2f1, E2f1 pro-apoptotic role is more evident in the absence of p53, where its function could be counteracted by E2f2. Therefore, E2f2 and Dp anti-apoptotic function is only revealed when p53 is not present (Wichmann et al., 2010).

Another factor implicated in the p53-independent apoptotic induction is the JNK pathway (McNamee and Brodsky, 2009) (Figure 1). Although, this pathway is activated in a p53-dependent manner at early time points (4 h post-IR), it also plays an important role regulating p53-independent cell death. The activation of the JNK pathway in response to IR, monitored by the expression of the JNK target gene *puc*, was observed in *p53* mutant discs at later time points, suggesting that the JNK pathway is also active in a p53-independent manner (McNamee and Brodsky, 2009). Functional studies have demonstrated that the levels of JNK signaling determine the amount of p53-independent apoptosis. In this sense, the ectopic expression of *puc* reduces apoptotic induction and Hid levels both at early and late time points (McEwen and Peifer, 2005; McNamee and Brodsky, 2009; Shlevkov and Morata, 2012). Accordingly, reducing the dosage of *puc* and therefore hyperactivating the JNK pathway in p53 mutant discs show an increase on caspase activation (McNamee and Brodsky, 2009).

In a genome-wide expression analysis for differentially expressed genes after exposure to IR, van Bergeijk et al identified the anti-apoptotic function of the elongation factor EF1-a 100E. Reducing the function of this factor increases the levels of cell death induced by IR only in p53-depleted cells (van Bergeijk et al., 2012).

It is important to note that the induction of p53-independent apoptosis after irradiation is observed at time points when wing discs cells have resumed their cell cycle. After irradiation, cells are transiently arrested in G2 in a Chk1/Grp dependent manner. This is due to the inhibition of the activity of Cdk1 through the kinase Myt1 (Fogarty et al., 1997; Jin et al., 2008). Although induction of apoptosis is progressively observed after this G2 stall, wing disc mutant for *Chk1/grp*, that failed to properly arrest, show an increase of JNK signaling and p53-independent apoptosis (McNamee and Brodsky, 2009). Using the *Minute* bristle phenotype as readout of segmental aneuploidy in the adult, Mcnamee and Brodsky propose that the activation of the JNK pathway limits the number of aneuploid cells induced by IR (McNamee and Brodsky, 2009). This segmental aneuploidy generated by DSB leads to the loss of ribosomal genes that eventually reduce protein synthesis, an event that has been demonstrated to trigger the elimination of unfit cells through cell competition. This is a process that strongly depends on JNK apoptotic induction (Moreno et al., 2002).

In summary, p53-dependent apoptosis contributes to maintain genetic stability through the fast and canonical DDR pathway. The function of p53 is not only required for the activation of the proapoptotic genes but also to repress the expression of anti-apoptotic factors such as Ef1a-100. Later on, a p53-independent apoptosis takes place after cells have recovered from the DNA damage cell cycle arrest to eliminate cells which are less fit through the activation of the JNK pathway and E2f1 that counterbalance the anti-apoptotic activities (Titen and Golic, 2008; McNamee and Brodsky, 2009; Wichmann et al., 2010). The relative simplicity of the *Drosophila* p53, JNK, and E2f families compared to vertebrates have been fundamental to dissect the relative contributions of these factors in the induction of apoptosis after DNA damage.

## Differential apoptotic sensitivities to DNA damage depending on proliferative status of the cell

Both in mammals and in *Drosophila*, the most responsive cells to DNA damage induced apoptosis are the ones that are actively dividing (MacCallum et al., 1996; Minter et al., 2002; Moon et al., 2005; Qi and Calvi, 2016; Kurtz et al., 2019; Ruiz-Losada et al., 2022). How cell cycle status affects apoptotic response after DNA damage is a fundamental question that still remains largely unknown. *Drosophila* has been an excellent biological model for establishing the basic mechanisms involved in this process.

### Cell cycle regulation of DNA-damage apoptotic induction

**FIGURE 2:**
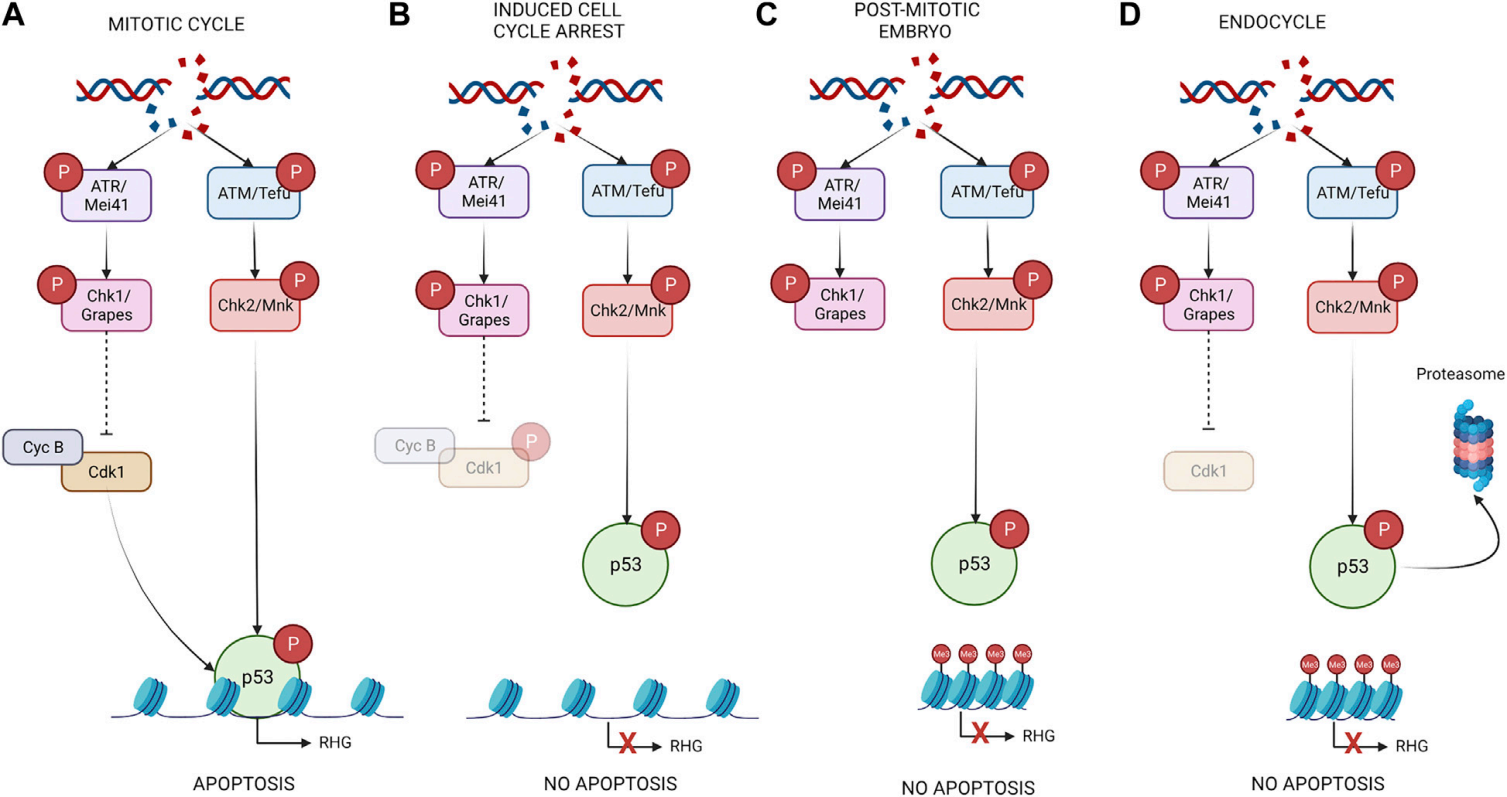
DNA damage can lead to different outcomes depending on the cell cycle stage of the cell. **(A)** In cycling cells, IR activates p53 through the DDR pathway. The presence of an active CycB/Cdk1complex facilitates the binding of p53 to the proapoptotic genes and therefore the induction of apoptosis. **(B)** In induced cell cycle arrested cells p53 is initially activated by DDR pathway, however since these cells do not have active Cdk1, p53 fails to induce the expression of *rpr* and *hid.* **(C)** In embryonic post-mitotic cells, the epigenetic silencing of the IRER interferes with the ability of p53 to regulate the expression of the proapoptotic genes. **(D)** In endocycle cells even though the DDR pathway is active in response to IR, apoptosis is not induced. This is due to at least two mechanisms of control. On one hand, an epigenetic silencing at the regulatory region of the proapoptotic genes blocks the ability of p53 to induce their expression. Secondly, the levels of p53 in these cells are significatively lower that in mitotic tissues through p53 targeted degradation by the proteasome. Created with BioRender.com.

When cycling cells of the imaginal discs are exposed to a genotoxic stress, like IR, the DDR pathway promotes a fast, but transient, G2 arrest, that is followed by apoptosis induction (Brodsky et al., 2000a; Sogame et al., 2003; Brodsky et al., 2004; Jaklevic and Su, 2004; Ruiz-Losada et al., 2022). This apoptotic induction is due to the activity of p53 that triggers the transcription of the proapoptotic genes as we have previously described (Brodsky et al., 2000a; Lee et al., 2003; Sogame et al., 2003; Brodsky et al., 2004; Ruiz-Losada et al., 2022) (Figure 2A). However, experimentally induced cell cycle arrest strongly compromises the ability of those cells to induce the apoptotic program (Qi and Calvi, 2016; Ruiz-Losada et al., 2022). This apoptotic inhibition in non-proliferating cells is also observed in developmentally arrested cells. For example, post-mitotic cells of the *Drosophila* head or the ZNC in the wing imaginal disc are refractory to IR-induced apoptosis (Moon et al., 2005; Kurtz et al., 2019; Ruiz-Losada et al., 2022). Similar response was described in *Drosophila* embryos, where the window of apoptotic sensitivity correlates with the timepoints when cells are highly proliferating (Zhang et al., 2015). Early-stage embryos (stage 9–11) are more sensitive to DNA damage apoptotic induction than older embryos (stage 13 and later). This sensitive to resistant transition correlates with the entry of most embryonic cells to post-mitotic differentiation (Zhang et al., 2015). In all these developmentally and experimentally arrested cells, the expression of the proapoptotic genes was not induced in response to DNA damage. Several mechanisms have been proposed to explain the differential apoptotic sensitivity of non-proliferating cells to irradiation. In wing imaginal discs, the G2/M promoting factor Cdk1 physically and genetically interacts with p53, facilitating the binding of p53 to the proapoptotic genes. Therefore, in experimentally arrested cells of the wing imaginal disc, active Cdk1 is not present preventing the regulation of *rpr* and *hid* by p53 (Ruiz-Losada et al., 2022) (Figure 2B). Accordingly, in post-mitotic cells of the head, p53 binding at the *rpr* p53^RE^ was lost (Kurtz et al., 2019). This molecular connection between p53 and Cdk1 links cell cycle progression to apoptotic induction in response to DNA damage.

In embryos an epigenetic control of chromatin accessibility at the irradiation responsive enhancer region (IRER), which contains the *rpr* p53^RE^, has been proposed to regulate the responsiveness of the proapoptotic genes to IR during the sensitive to resistant transition (Zhang et al., 2008; Zhang et al., 2015) (Figure 2C). This silencing would interfere with the ability of p53 to regulate the expression of proapoptotic genes in response to IR. An important question is how the cell cycle machinery trimethylates H3K27/H3K9 specifically at the proapoptotic locus to form a heterochromatin-like structure during the sensitive-to-resistant transition (Figure 2C). Interestingly, chromatin accessibility at the *rpr* and *hid* loci was not affected in experimentally arrested wing imaginal cells, suggesting that multiple mechanisms could regulate the sensibility to IR-induced apoptosis in proliferating and arrested cells (Ruiz-Losada et al., 2022).

The molecular connection between the cell cycle and the apoptotic pathway could help the damaged cells to activate the appropriate responses after genotoxic stress. Therefore, when cells with damaged DNA are transiently cell cycle arrested, p53 proapoptotic function is inhibited allowing the DNA repair mechanisms take place. However, if the DNA lesions are not repaired and cells have resumed cell cycle progression, p53 proapoptotic activity helps eliminate the damaged cells (Figure 2A). In human cells, Cdk1/Cdc2 phosphorylates p53 at serine 315 promoting p53 binding site preference, target selection and transcription stimulation (Bischoff et al., 1990; Wang and Prives, 1995; Blaydes et al., 2001). It is possible that this mechanism could be employed by p53 in response to DNA damage to regulate different transcriptional outputs depending on the proliferation status of the cell.

Recent reports have shown that tissue injury in the imaginal cells promotes a cellular stress that activates the JNK pathway to induce a transient G2 stalling through the downregulation of the Cdk1 activator, String (Cdc25). As the JNK pathway is an important apoptotic trigger, it has been proposed that this cell cycle arrest protects cells from JNK-induced cell death while inducing proliferative signals to the surrounding tissue during wound healing (Cosolo et al., 2019). A similar cell cycle arrest mechanism could be employed by cells to cope with different stress stimuli to prevent apoptosis.

In response to DNA damage, mammalian cells could also enter in a permanent cell cycle arrest known as senescence where the activation of p53 and the Cdk inhibitor p21 play prominent roles (Kumari and Jat, 2021). Remarkably, senescent cells are protected from DNA-damage induced apoptosis (Childs et al., 2014). It would be interesting to investigate whether any of the mechanisms described above could be employed by senescent cells to prevent apoptotic induction.

### Endocycle cells are protected from DNA-damage induced apoptosis

Some *Drosophila* tissues such as the salivary gland, the fat body, or the follicle cells and germ cells from the germarium enter a modified cell cycle known as the endocycle. During this process, G and S phases alternate without entering mitosis through the downregulation of Cdk1 activity (Edgar et al., 2014). In these developmental endocycle cells, the DNA replication stress has been found to trigger the DDR pathway without inducing apoptosis (Mehrotra et al., 2008; Zhang et al., 2014). Moreover, the endocycle cells are resistant to IR, as they have very limited ability to activate the proapoptotic genes (Mehrotra et al., 2008; Zhang et al., 2014; Ruiz-Losada et al., 2022) (Figure 2D). When looking at the levels of p53, Zhang et al found a clear difference between cycling cells of the imaginal discs and the endocycling cells of the salivary glands and the fat body (Zhang et al., 2014). While p53 protein is readily detected in imaginal cells and is able to bind to the *rpr* and *hid* p53^REs^, significant lower levels where found in endocycling tissues where no p53 was bound to the proapoptotic genes (Zhang et al., 2014) (Figure 2D). Moreover, ectopic expression of *p53* in endocycling cells is not sufficient to induce apoptosis, suggesting that other mechanisms may regulate the apoptotic competence of those cells (Mehrotra et al., 2008; Zhang et al., 2014). Again, an epigenetic silencing was found in endocycling cells at the proapoptotic genes *rpr* and *hid* that blocks the ability of p53 to induce their expression (Zhang et al., 2014) (Figure 2D).

Similar apoptotic resistance to DNA damage was found when mitotic cells of the wing imaginal discs were forced to enter the endocycle by the expression of the Anaphase-Promoting Complex/Cyclosome (APC/C) binding protein Fizzy-related (Fzr/Cdh1) (Ruiz-Losada et al., 2022). The APC/C complex mediates the degradation of all the mitotic cyclins, preventing Cdk1 activation and therefore mitotic entry. In these imaginal endocycle induced cells, no changes were found in p53 protein levels and chromatin accessibility at the proapoptotic loci, suggesting that other mechanisms prevent apoptotic induction (Ruiz-Losada et al., 2022). As the endocycle suppresses Cdk1 activity, it is possible that in these cells p53 ability to bind and activate the expression of the proapoptotic genes is suppressed due to the lack of active Cdk1, as we have described above (Ruiz-Losada et al., 2022) (Figure 2B). This is in contrast to polyploid cells of the ovary and the salivary glands, in which forced expression of *CycA* and *Cdk1* do not confer them apoptotic competence, even when these cells inhibited the endocycle program and enter in mitosis (Qi and Calvi, 2016). Therefore, developmentally induced endocycle cells could employ a built-in genetic program to block apoptotic response to replication stress and DNA damage through the epigenetic silencing of the proapoptotic genes. These results highlight the different mechanisms that cells could use to repress IR-induced apoptosis.

## Differential responses to DNA damage depending on cellular context

In contrast to the imaginal discs, very little apoptosis was observed in other tissues of larvae exposure to IR, including the gut, brain, lymph gland, ring gland, salivary glands, and fat bodies (Halme et al., 2010). Some of these tissues (salivary glands*,* fat body) are composed mainly for polyploid cells that undergo endocycles that, as we have described above, is a process that blocks the activation of the apoptotic pathway. However, cells that are actively proliferating and that form part of organs, such as the gut and the brain have been reported to be radioresistant. One important feature of these structures is that both contain stem cells. To maintain genomic stability after DNA damage and to avoid the transmission of mutations into progenitor cells, stem cells also activate a coordinate response, as we have described in wing discs (Adams et al., 2010; Serrano et al., 2011). It has been reported that compared to their derived progenitors, stem cells show a higher expression of genes involved in DNA damage signalling and checkpoints. When DNA damage cannot be repaired, cells undergo senescence, apoptosis or differentiation. The specific response triggered by genotoxic stress largely depends on the stem cell type and their developmental context (Mujoo et al., 2016).

### Germinal stem cells

**FIGURE 3:**
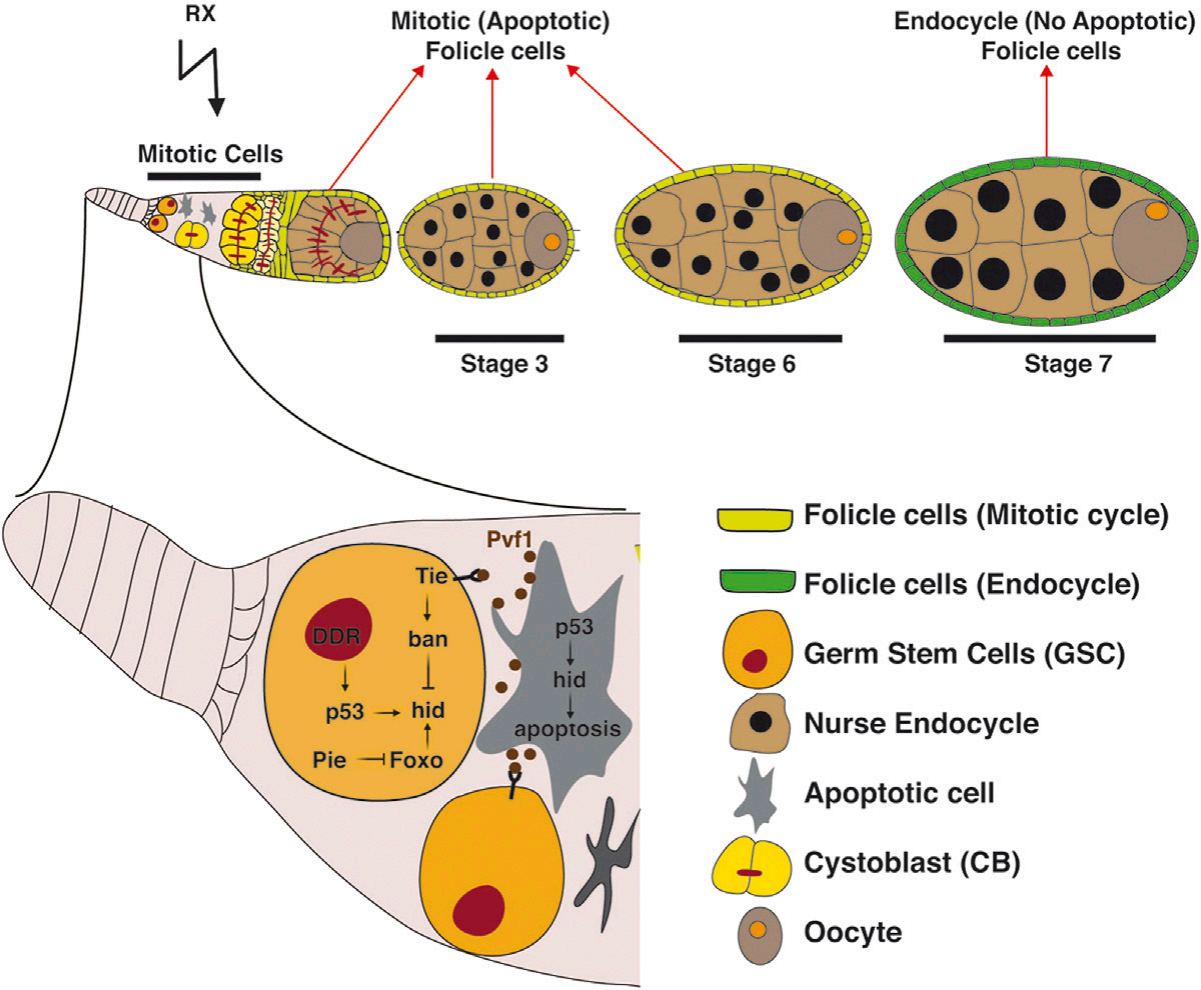
A schematic representation showing the germarium of the *Drosophila* ovary. Proposed mechanism of how female GSCs are protected against apoptosis through the collaborative contribution of multiple signalling pathways. Dying cells send Pvf1 as a survival signal that activates the Tie receptor in GSCs. Tie activation leads to *ban* microRNA upregulation that represses Foxo and p53 mediated *hid* activation in response to DNA damage. Created with BioRender.com.

Oocytes arise from two to three ovarian germline stem cells (GSCs). These cells are located at the anterior end of the germarium, adjacent to the niche cap cells (CpCs). The GSC divides asymmetrically generating a GSC and a transit-amplifying (TA) daughter cystoblast (CB). Cystoblasts divide four times with incomplete cytokinesis, forming cysts of 16 interconnected germline progenitors. In each cyst, only one cell will form the oocyte and will initiate meiosis, while the other 15 will enter the endocycle and will differentiate as polyploid nurse cells (Lin, 2002; McLaughlin and Bratu, 2015). The germline cysts are surrounded by a population of somatic cells that proliferate by mitotic divisions until stage 6, and then switch into endocycles in response to Notch signaling (Hassel et al., 2014). The linear arrangement of oocyte production in the ovary facilitates the observation of the proliferative stages of GSCs and their progeny, as well as the spatiotemporal regulation of meiosis (Figure 3).

In the *Drosophila* male, the stem cell niche is located at the blind apical end of the testis. Post-mitotic somatic hub cells comprise a key component of this niche, supporting 10 to 12 GSCs (de Cuevas and Matunis, 2011). GSCs divide asymmetrically to generate one cell that remains a stem cell and a daughter cell known as gonialblasts (GBs). This cell is enveloped by two somatic cyst cells, which arise from cyst stem cells (CySCs) that also divide asymmetrically to self-renew. As in the female germline, GBs divide four times with incomplete cytokinesis to generate a cyst of 16 spermatogonial cells, which enter premeiotic S-phase shortly after their last mitotic division. These 16 cells remain connected by stable intercellular bridges called ring canals (de Cuevas and Matunis, 2011).

In ovaries, IR treatment induces cell cycle arrest in S and G2 phases in the mitotically dividing germline cells in a Chk2/Mnk and Chk1/Grp dependent manner. The ATR/Mei41 kinase is only required for the S-phase checkpoint, but its function is not necessary for the G2/M checkpoint (Shim et al., 2014). The S-phase checkpoint observed in these cells contrast with the observation that in wing discs IR only induces cell cycle arrest in G2 (see above) (Ruiz-Losada et al., 2022). In eukaryotic cells, DNA damage can trigger the activation of the intra-S checkpoint during S phase to protect genomic integrity and ensure replication fidelity. This checkpoint is induced when replication forks are stalled and is mediated by the function of ATR (Iyer and Rhind, 2017). Therefore, unlike wing cells, in GSCs the intra-S checkpoint seems to be required to prevent genomic instability.

In the germarium IR exposition induces cell death in mitotically dividing somatic cells, but not in endocycling follicle and nurse cells and GSCs (Figure 3) (Park et al., 2019; Hassel et al., 2014; Shim et al., 2014; Xing et al., 2015). Apoptosis of TA cells results in the loss of differentiating multi-cell cysts. This effect is only transitory, since 7 days post-IR treatment multi-cell cysts are observed again, indicating that the irradiated GSCs are able to generate new cells (Xing et al., 2015). Similar to female GSCs, irradiated testes also show a rapid loss of spermatocyte cysts while the GSCs are not affected. Therefore, GSCs in ovaries and testes are more resistant to IR-induced apoptosis than their differentiating progenies (Wylie et al., 2014; Hasan et al., 2015; Xing et al., 2015). The apoptosis detected in both, germarium, and testis, is p53 and Chk2/Mnk dependent and it is the cause of the loss of differentiating multi-cell cysts within 3 days after exposure (Shim et al., 2014). The irradiation-induced cell death in the germarium occurs through p53-dependent transcriptional activation of the *hid* gene (Park et al., 2019).

Surprisingly, IR exposure activates p53 selectively in GSCs and their immediate daughters, although these cells do not undergo apoptosis (Wylie et al., 2014). Therefore, in these cells there is not a clear connection between p53 status and apoptosis. These results imply that the pro-apoptotic function of p53 is blocked in GSCs. Since these stem cells are actively proliferating, the inability of p53 for inducing apoptosis cannot be due to the absence of active Cdk1, as we previously described in cell cycle arrested cells (Qi and Calvi, 2016; Ruiz-Losada et al., 2022). Another interesting observation is that in irradiated *p53* mutants, the re-entry in the cell cycle of the GSCs was significantly delayed compared to non-irradiated cells (Wylie et al., 2014). This result suggests that p53 might be involved in regulating cell cycle progression in these stem cells; surprisingly, this function would be required for promoting proliferation.

Apoptosis is attenuated in GSCs through joint efforts from multiple signalling pathways (Figure 3). The microRNA *bantam* (*ban*) is highly expressed in GSCs and its function is required for maintenance of these cells (Shcherbata et al., 2007; Xing et al., 2015). A previous report has shown that in wing discs this microRNA represses IR-induced apoptosis through targeting the 3′-UTR of *hid* mRNA (Jaklevic et al., 2008). The activation of *ban* depends on the receptor tyrosine kinase of the VGFR/PDGFR family encoded by the gene *tie.* In response to IR exposure, the apoptotic cells express the ligand Pvf1, which non-autonomously activates Tie and *ban* in adjacent cells, and as a consequence the function of hid is repressed in these cells. This process prevents further IR-induced apoptosis in the surrounding cells (Bilak et al., 2014). Interestingly, this mechanism is conserved in the germarium (Figure 3). After irradiation the levels of Pvf1 are significantly elevated in the transit-amplifying cells, especially in the cystoblasts, while are undetectable in the GSCs. Therefore, Pvf1 from the dying daughter cells would activate Tie in GSCs to upregulate *ban* microRNA and consequently repress *hid*, thereby protecting GSCs against IR-induced apoptosis (Xing et al., 2015). In addition to this mechanism, it has been described that the JAK-STAT pathway also plays a key role in the testis stem cells promoting stem cell survival by up-regulating the expression of the antiapoptotic factor Diap1 (Hasan et al., 2015). This signalling pathway activates, directly or indirectly, the expression of *Diap1* in the GSCs and CySCs. Therefore, the down-regulation of JAK–STAT signalling sensitizes the stem cells to IR-induced apoptosis. These results support a model in which Diap1 is required for survival of GSCs and CySCs in the testis niche, and attenuates the impact of death-inducing stimuli, such as IR (Hasan et al., 2015).

It has been described other factors that are involved in the response of GSCs to IR. The *Drosophila* ortholog of human G2E3 ubiquitin ligase, *pineapple eye* (*pie*) was identified as a cell survival factor in a screen searching for essential genes in stem cell self-renewal (Xing et al., 2012). The depletion of *pie* causes apoptosis of somatic cells in the testis and the ovaries but not in GSCs. The cell survival role of *pie* in somatic tissue is through the regulation of the level of the insulin pathway transcription factor Forkhead box O (Foxo). As an E3 ligase that ubiquitinates proteins, Pie targets Foxo for degradation. Studies in larval eye discs showed that DNA damage-induced apoptosis in response to UV irradiation is mediated by JNK/Foxo signalling, in which Foxo and Kay/Fos transcriptionally activate the proapoptotic gene *hid* (Luo et al., 2007). Accordingly, the reduction of *pie* induces high levels of Foxo that in turn increases the expression of *hid* triggering apoptosis of differentiating cells (Xing et al., 2015). Pie also targets Foxo in GSCs, although these cells do not die in mutant *pie* (Figure 3). This is due to the function that *ban* plays repressing *hid* in GSCs (see above). Importantly, in *foxo* mutants IR exposure causes a strong reduction in the number of GSCs, suggesting that the correct Foxo levels are critical for stem cell survival (Xing et al., 2015).

### Intestinal stem cells

The *Drosophila* intestinal epithelium is a monolayer composed of three types of cells; intestinal stem cells (ISCs), enterocytes (ECs), and enteroendocrine (EE) cells (Micchelli and Perrimon, 2006). The fly and human intestines share similar tissue, anatomy, and physiological function (Leulier and Royet, 2009). *Drosophila* gut homeostasis is maintained by replacing damaged cells with new ones that are generated from ISCs (Ayyaz and Jasper, 2013). In response to damage, ISCs divide asymmetrically to generate a population of non-differentiated and non-mitotically active progenitors known as enteroblasts (EBs) (Ohlstein and Spradling, 2006). The levels of Notch signalling activity define whether these cells differentiate into either an EC or an EE cell (Ohlstein and Spradling, 2006; Biteau et al., 2011; Perdigoto et al., 2011; Cordero and Sansom, 2012; Kapuria et al., 2012). After exposure to IR, the DNA damage marker γ-H2av foci increases in most cells of the guts, except in ISCs and its daughter cells, EBs. However, a fraction of ISCs and EBs undergo apoptosis. Accordingly, the levels of expression of the proapoptotic genes, *hid* and *rpr* increases in these cells upon irradiation when compared to non-irradiated controls (Sharma et al., 2020). Surprisingly, the expression of the reporter of JNK signalling *puc*, is not altered in ISCs, suggesting that the JNK signal is not active upon irradiation in these stem cells (Sharma et al., 2020). The fraction of ISCs that undergo apoptosis is very small compared to the large proportion of EE and EC cells that die, indicating that these stem cells are also resistant to IR-induced apoptosis (Xing et al., 2015). Although some of the signaling pathways involved in the maintenance and proliferation of GSCs are conserved in ISCs (see below), it is not clear if all the mechanisms described in GSCs for attenuating IR-induced apoptosis also operate in ISCs. It will be interesting to examine whether different stem cell types share a similar protective mechanism.

Irradiated flies contain intestines that are shorter and more permeable than non-irradiated controls, which indicate that irradiation structurally damages this tissue (Sharma et al., 2020). This effect is also observed in mammals and even in patients receiving radiation or chemotherapy (Poglio et al., 2009; Yu, 2013; Koukourakis, 2012). IR not only increases apoptosis in the midgut but also affects the proliferative ability of ISCs. Thus, as early as 1 day after IR exposure ISCs proliferation is inhibited (Sharma et al., 2020). When ISCs proliferation is forced in irradiated guts, through the over-expression of cell cycle regulators, such as CycE, the radiation-induced intestinal permeability is partially restored, and the intestinal barrier function is improved. These results suggest that IR induces the apoptosis of some ISCs, and reduces its proliferative potential in others. The combination of both effects reduces the population of ISCs, which decreases the regenerative ability of the intestine (Sharma et al., 2020). Similarly to the GSCs in the ovary, the gene *pie* is specifically required for ISCs maintenance by Foxo control of cell division, but not through apoptotic cell death (Xing et al., 2015).

In a genome-wide association study (GWAS) for radiation-induced intestinal permeability in *Drosophila*, the RNA binding protein, Musashi (*msi*) was identified as one of the possible genes associated with changes in intestinal permeability upon radiation (Sharma et al., 2020). The function of *msi* is specifically required for ISCs proliferation in irradiated, as well as in non-irradiated ISCs. The down-regulation of this gene in ISCs followed by irradiation enhanced the defect in gut permeability. However, *msi* overexpression induces intestinal stem cell proliferation, which partially restores radiation-induced intestinal permeability. Interestingly, *msi* role seems to be evolutionary conserved, since the human orthologue *msi1*, is strongly expressed in the intestinal crypts, especially during embryonic development and regeneration (Potten et al., 2003).

### Brain neuroblasts stem cells

*Drosophila* larval brain contains neural stem cells called neuroblasts (NBs). These cells are specified during the embryogenesis and divide asymmetrically to produce differentiated neurons and glial cells. Considering the lineage of brain NBs, it is possible to distinguish two different types: the NBs with a type I lineage, and neuroblasts with type II lineage. The NBs type I, that are the most abundant in the central brain (approximately 180 NBs), undergo stem cell-like asymmetric divisions, to self-renew and to generate two smaller daughter cells known as ganglion mother cells (GMCs). These cells divide only once to give rise to two post-mitotic neurons and/or glial cells. The NB type II are much less abundant (18 NBs), and divide asymmetrically to self-renew and generate intermediate neural progenitors (INPs). INPs have the capacity of undergoing up to 10 rounds of asymmetric division to self-renew and to generate GMCs and neurons throughout larval development. At the end of the late third instar larvae or early pupal stages, NBs stop proliferating and exit the cell cycle (Urbach and Technau, 2004; Bello et al., 2008; Boone and Doe, 2008).

Exposition of larval brains to IR during the first instar larval stage causes microcephaly, although neither the NBs nor the differentiating cells undergo apoptosis (Halme et al., 2010; Wagle and Song, 2020). Larvae irradiated at later stages do not show any effect in brain growth (Poodry and Woods, 1990; Halme et al., 2010). Therefore, microcephaly cannot be due to an excess of cell death. It remains unknown the mechanisms that protect NBs from IR-induced apoptosis, this is an interesting question to be addressed.

Ionizing radiation affects NBs cell cycle progression and proliferation. Compared to control brains, irradiation reduced the number of NBs in S phase, as early as 1 h after IR treatment (Jaklevic and Su, 2004). These results suggest that irradiation promotes inhibition of G1-S transition and/or slowing down the ongoing S phase. This result is consistent with the activation of an intra-S checkpoint, as we previously described in the mitotically dividing germline cells. The function of ATR/Mei41 and Chk1/Grp is necessary for the control of this checkpoint (Jaklevic and Su, 2004). Interestingly, similar results have been obtained in mammalian cells (Falck et al., 2001; Zhao et al., 2002). Irradiation also affects the entry into mitosis of NBs. When late-third-instar larvae were exposed to increasing doses of IR the mitotic index of NBs dropped progressively, indicating a delay in initiating mitosis (Royou et al., 2005; Wagle and Song, 2020). This effect was not observed in *Chk1/grp* mutant brains, demonstrating the requirement of this signal to delay entry into mitosis in response to irradiation (Royou et al., 2005). Interestingly, the function of Chk1/Grp is also necessary in NBs during mitosis to delay anaphase onset (Royou et al., 2005). This function is exerted in parallel with spindle-checkpoint components, such as *bubR1*. Thus, in irradiated brains the number of mitotic cells in anaphase is reduced compared to control untreated brains. Similarly, this effect is also observed in irradiated mutant larval brains for *Chk1/grp* and *bubR1*, but no in *Chk1/grp bubR1* double mutants in which the frequency of mitotic cells in anaphase is comparable to that observed in control untreated brains (Royou et al., 2005).

The smaller brain size induced by IR cannot be caused solely by the transient effects on the cell cycle on NBs (Wagle and Song, 2020). It has been shown that proliferation of NBs at later time points was also significantly reduced after irradiation compared to non-irradiated animals (Wagle and Song, 2020). This effect on NBs proliferation is caused by the premature differentiation of NBs. During brain development, the end of NBs proliferation and the induction of terminal differentiation is triggered by the nuclear translocation of the transcription factor Prospero (Pros) (Choksi et al., 2006). In irradiated brains the percentage of NBs with nuclear Pros strongly increases 48 h after IR treatment. These data suggest that irradiation of larvae at early third instar stage induces premature differentiation of NBs, resulting in NBs loss and a subsequent retardation of brain growth (Wagle and Song, 2020). Therefore, in response to IR, NBs activate signals to induce premature differentiation preventing the proliferation of stem cells with genomic alterations (Wagle and Song, 2020). Aneuploidy also causes brain size reduction due to a decrease in the number of proliferative neural stem cells (NSCs), but not through apoptosis (Gogendeau et al., 2015).

### Stem-like cells in the wing disc

**FIGURE 4:**
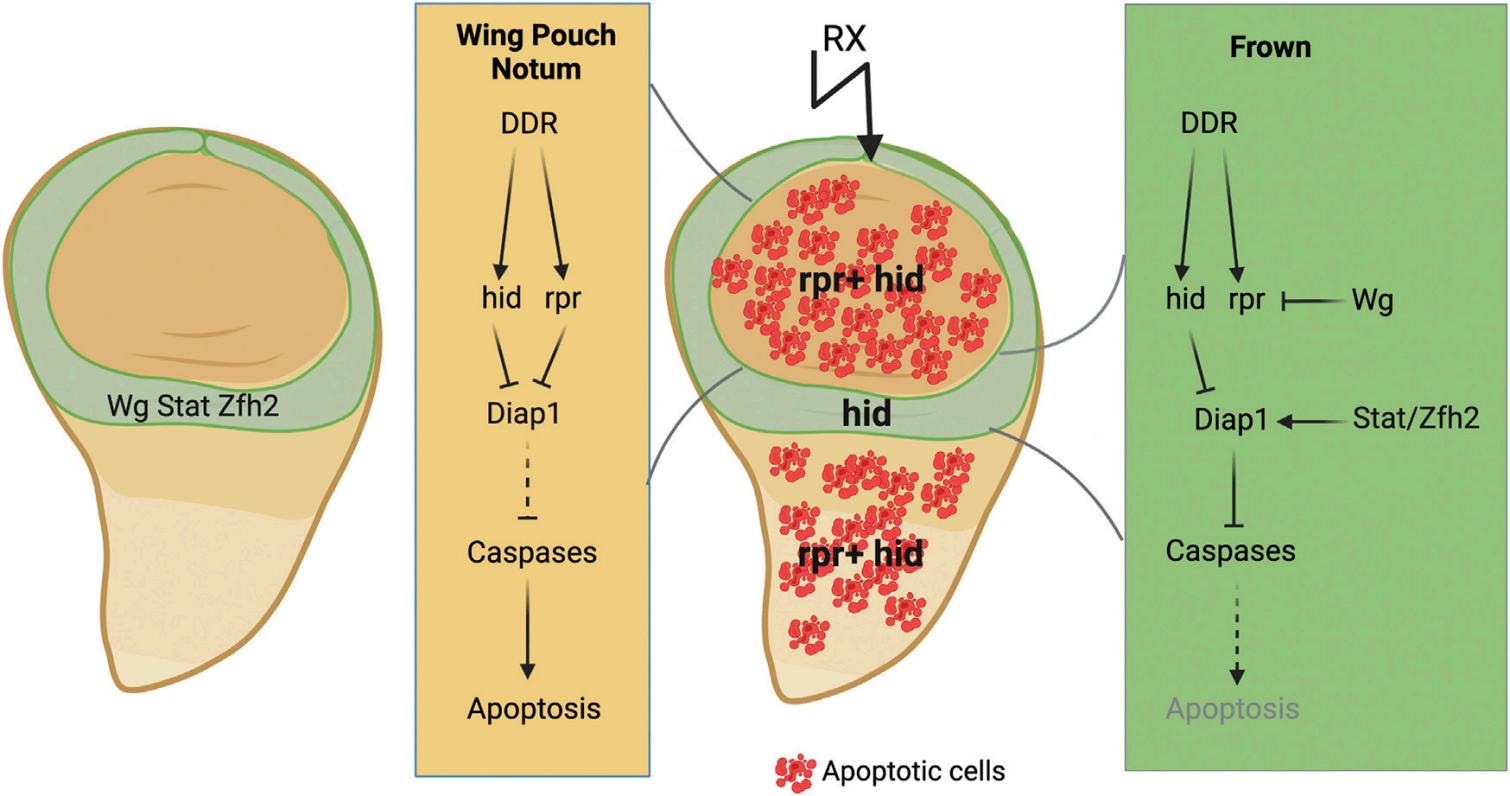
A schematic diagram showing the different responses after IR in the three main parts of the wing disc. In green is indicated the frown (hinge). In this region Wg and Stat signalling are strongly activated. The function of these signalling pathways in this domain prevents the induction of *rpr* in response to IR, and therefore apoptosis. Created with BioRender.com.

Different studies have shown that within the wing imaginal disc exist different domains of cells with different sensitivity to undergo apoptosis in response to IR (Moon et al., 2005; Verghese and Su, 2016; Ruiz-Losada et al., 2022). IR-induced apoptosis was reproducibly robust in the wing pouch, excepting some regions such as ZNC (see above), whereas it was consistently low in the dorsal part of the future wing hinge, known as the frown (Verghese and Su, 2016) (Figure 4). In contrast to other regions of the wing discs, the over-expression of *p53* in the hinge was not sufficient for inducing cell death, indicating that the cells in this domain are resistant to apoptosis (Zhang et al., 2014). The cells in the hinge region express high levels of *wingless* (Wg, *Drosophila* Wnt-1) and *Stat92E* (the sole STAT gene in *Drosophila*) (Figure 4). Depletion of the function of *Stat92E* or *wg* increases IR-induced apoptosis in the dorsal hinge. The combined down-regulation of both genes did not augment the apoptotic effects induced by individual mutant conditions of each gene, suggesting that STAT and Wg function in a single pathway or they act on the same target genes. However, this epistasis analysis was carried out using hypomorphic conditions, so it is not conclusive. These data indicate that IR-resistance in the dorsal hinge requires Wg and STAT (Verghese and Su, 2016) (Figure 4).

IR-induced apoptosis depends on the transcriptional activation of the proapoptotic genes, specifically of *hid* and *rpr*, which antagonize Diap1 to allow caspase activation (Brodsky et al., 2000a; Lee et al., 2003; Sogame et al., 2003; Goyal et al., 2000) (Figure 1). While the expression levels of *Diap1* and *hid* are not different between the pouch and the frown in irradiated and non-irradiated discs, *rpr* expression shows a clear difference. While *rpr* expression*,* visualized by the activity of an 11-kb fragment upstream of the transcription start, is low in the frown in non-irradiated discs, it increases throughout the discs after irradiation, excepting in the frown (Figure 4). This observation could explain the differences in IR sensitivity. The down-regulation of Wg signalling in irradiated discs causes the up-regulation of *rpr* in the frown, an effect, that is, consistent with the function of Wg blocking IR-induced apoptosis in this region (Figure 4). However, the depletion of Stat92E has little effect on *rpr* expression, implying that the function of STAT preventing IR-induced apoptosis is likely through another target besides *rpr* (Verghese and Su, 2016). This could be due to the direct regulation of *Diap1* by the transcriptional effector Stat92E (Betz et al., 2008). The function of JAK-STAT would help to maintain a threshold of Diap1 levels that Hid, Rpr, and Skl must collectively overcome.

Interestingly, it has been proposed that upon damage or IR-induced apoptosis, cells in the wing hinge acquire stem cell-like properties, such as the ability to change cell fate, and to translocate to another region of the disc (Verghese and Su, 2016; Verghese and Su, 2017).

The transcription factor Zfh2 is an effector in the JAK/STAT pathway, and plays a key role in the induction of stem cell-like behaviour of the cells of the frown (Figure 4). During development, the expression of this factor is confined to the hinge and its function is necessary for the development of this region (Terriente et al., 2008). Zfh2 is also involved in preventing IR-induced apoptosis of the cells of the hinge (Verghese and Su, 2016; Verghese and Su, 2018). Moreover, autonomous depletion of Zfh2 in irradiated discs inhibited fate change and translocation of the cells of the hinge (Verghese and Su, 2018).

The induction of the regenerative behavior of the cells of the frown in response to damage is mediated by caspase activity (Verghese and Su, 2018). Considering these findings, Verghese S and Su TT have proposed a model to explain how the combined activity of several proapoptotic genes define the different responses that are generated in wing discs following irradiation (Verghese and Su, 2018) (Figure 4). Thus, after irradiation, *hid* expression increases throughout the disc, whereas the expression of *rpr* is induced in the notum and the pouch, but not in the cells of the hinge, where it is repressed by Wg signalling. The co-expression of *hid* and *rpr* in the cells of the notum and the pouch results in increased apoptosis, while the expression of *hid* alone in the hinge cells is insufficient for activating caspase to induce apoptosis, but sufficient for promoting fate change and translocation. The activity in the frown of Zfh2 and STAT/Wg would maintain low level of effector caspase activity after irradiation in these cells, thereby allowing them to acquire new fate and relocate (Verghese and Su, 2018).

The existence of a subpopulation of epithelial cells that are intrinsically resistance to apoptosis has been linked to the regenerative ability shown by the wing discs (Verghese and Su, 2016). The apoptotic-resistant cells residing in the hinge region would survive radiation exposure or other damage insults, which will allow them to proliferate and participate in the regeneration of the organ. It has been reported that cells of the frown participate in rebuilding the wing pouch but not vice versa. This regenerative model does not rely on the presence of a specialized type of stem cells. Instead, differences in gene expression can create a subpopulation of cells that fulfil this function (Verghese and Su, 2016) (Verghese and Su, 2017).

## Conclusion

Much is known about the activity of the components of the DDR pathway controlling the different cellular responses after DNA damage. However, how these responses are regulated in different cellular contexts is less explored. The mechanisms that control cell proliferation and apoptosis in response to genotoxic stress must be tightly coordinated to maintain genomic integrity and tissue homeostasis. The existence of in-built mechanisms that might attenuate or block DNA-damage induced apoptosis in different cell types and cell proliferation states may well be informative for the clinical responsiveness of tumor cells to radiation-therapy. The lack of knowledge about this issue, therefore represents a very substantial gap in our understanding of the underlying cellular mechanism implicated in radioresistance of tumor cells. *Drosophila* is a powerful model system for analyzing the mechanisms that control cell behavior in the context of the complex interactions that take place between the different cell types that constitute an organism. Importantly, the basic signalling pathways and their regulation are highly conserved between flies and humans. Here we have revised the current state of knowledge of the mechanisms that modulate and coordinate the DNA damage responses in different cellular contexts in *Drosophila*. The different studies carried out in this organism have shown that the sensibility to IR-induced apoptosis depends on the proliferation state of the cells. They also have provided new insights about the intrinsic mechanisms that attenuate the apoptotic pathway in response to DNA damage in different stem cells. This effect seems to be through joint efforts from multiple signalling pathways.

### Future directions

The specific response triggered by IR exposition largely depends on the developmental context, the cell type and the proliferation status of the cell. The action of different signalling pathways in each cellular context can modulate the activity of the DDR pathway, which can lead to different outcomes. Therefore, it is not only important to characterize which members of the DDR pathway function in different cellular contexts and how they are coordinated, but also which signals can regulate its response. This is key to identify the mechanisms that modulate the sensibility to IR-induced apoptosis in distinct cellular contexts and therefore for understanding the underlying mechanism implicated in radioresistance of tumor cells.

High-throughput sequencing techniques have revealed that each tumor type typically exhibits distinct constellations of genetic alterations that can affect signalling pathway activity related with different aspects of cancer behavior. An important challenge is to associate these genetic changes with the specific features show by different tumor cells.

*Drosophila* provides a powerful *in vivo* model system for analyzing the signalling network that might be modulating the DDR in different cellular contexts and therefore the specific responses triggered by this pathway in each scenario. The knowledge generated in *Drosophila* can be used to gain new insight into the mechanisms that might be involved in modulating the DNA damage responses in different cellular contexts. These data can contribute to characterize the functional relationship between the genetic alteration exhibited by different tumor types, which have been defined through high-throughput technology, and the underlying cellular mechanisms implicated in radioresistance of tumor cells. The combination of multidisciplinary approaches might ultimately facilitate the development of personalized medicine.
